# Noise-induced Hearing Loss Among Dental Personnel in a University Hospital: Occupational Noise Exposure and Lifestyle Factors — A Cross-sectional Analytical Study

**DOI:** 10.1016/j.shaw.2026.02.002

**Published:** 2026-02-19

**Authors:** Thitiworn Choosong, Sittichok Teawanan, Ramida Dindamrongkul, Wandee Khaimook, Sasithorn Srimeechai, Paranicha Bunruang, Phattaranun Phiboon, Wannadee Wacharachaipong

**Affiliations:** 1 Department of Family and Preventive Medicine, Faculty of Medicine, Prince of Songkla University, Songkhla, Thailand; 2 Department of Oral and Maxillofacial Surgery, Faculty of Dentistry, Prince of Songkla University, Songkhla, Thailand; 3 Department of Otolaryngology Head and Neck Surgery, Faculty of Medicine, Prince of Songkla University, Songkhla, Thailand; 4 Faculty of Public Health, Thammasat University, Pathumthani, Thailand; 5 Faculty of Medicine, Prince of Songkla University, Songkhla, Thailand

**Keywords:** Dental personnel, Health behavior, Hearing loss, Occupational noise exposure, Risk factors

## Abstract

**Background:**

Sensorineural hearing loss is influenced by multiple factors, with occupational noise exposure being the key contributor. Dental personnel are routinely exposed to high-frequency sounds from clinical instruments. Although average clinic noise levels are typically below the 85 dBA threshold, cumulative exposure and high peak levels may increase the risk of noise-induced hearing loss (NIHL). This study assessed the workplace noise characteristics, prevalence, and risk factors for NIHL among dental personnel.

**Methods:**

This cross-sectional study was conducted with 184 staff members at a university dental hospital. Data collection included questionnaires, otoscopic examinations, pure-tone audiometry, and workplace noise measurements.

**Results:**

Hearing loss was identified in 30.4% of the participants. Individuals over 30 years of age and those who consumed tobacco and/or alcohol had significantly higher risks (odds ratio = 2.93, 5.81, and 3.21, respectively), whereas caffeine consumption showed a protective association. Technical and maintenance staffs were exposed to higher peak and high-frequency noise than clinical or administrative staff, with 8-h time-weighted averages ranging from 64.3 to 68.9 dBA—all below occupational noise exposure limits.

**Conclusion:**

Occupational noise exposure and lifestyle factors contributed to NIHL among dental personnel. Preventive strategies should integrate engineering controls to reduce peak noise alongside health promotion targeting modifiable risks such as tobacco and alcohol use. Early detection and implementation of hearing preservation strategies are essential to reduce the prevalence of NIHL in dental professionals.

## 1 Introduction

Hearing loss is a global health concern, and more than 5% of the global population lives with disabilities associated with hearing impairment [1]. Sensorineural hearing loss (SNHL) is the most common, caused by damage to the cochlea, auditory nerve, or central nervous system. The SNHL is attributed to multiple risk factors including occupational noise exposures. Excessive noise exposure is significant because it primarily damages the outer hair cells [2]; this damage initiates auditory dysfunction that leads to noise-induced hearing loss (NIHL). The NIHL is progressive and permanent as cochlear hair cells cannot regenerate, resulting in irreversible auditory impairment. Although NIHL has been identified as a preventable occupational disease, it remains highly prevalent across various sectors including industrial manufacturing and transportation.

The reported incidence of NIHL in Thailand was 15.6 cases per million people in 2023 [3]; however, this is likely underestimated owing to limited hearing screening services and insufficient awareness of occupational noise risks outside industrial sectors. Notably, most related research has focused on factory, mining, and construction workers, whereas healthcare professionals have received less attention in research and public health policies. However, healthcare professionals are regularly exposed to loud instruments that place them at an increased risk of developing NIHL [4]. Evidence from hospitals indicates that noise exposure can adversely affect both hearing and general health, although average noise levels remain below the occupational exposure limits [[5], [6], [7], [8]]. This lack of recognition has contributed to the underestimation of NIHL risk among healthcare professionals and delayed prevention programs.

Dental professionals are routinely exposed to prolonged periods of loud high-frequency sounds generated by instruments. This exposure has been associated with communication difficulties, reduced concentration, and elevated hearing thresholds [5]. A systematic review found dental occupations linked to hearing loss, with work experience as the main risk; dental students showed 4.17–5 dB greater threshold shifts than nondental students [9]. Although overall noise levels in dental clinics remain below 85 dBA, as recommended by the World Health Organization [[6], [7], [8]], specific equipment, such as air blow pipes and model trimmers, can generate noise levels exceeding this limit [6]. Additionally, repeated exposure and cumulative noise increase the overall noise level, leading to a significant risk of hearing threshold shifts [10]. In a previous study, dental hygienists who frequently used ultrasonic sealers showed a greater risk of developing NIHL than those who used them less often [11]. Almost studies mention on dental personnel [5,10,11] but have not included the dental students, who have a long period of laboratory and clinical rotation [12]. Existing evidence shows that NIHL risk is influenced not only by average noise levels but also by cumulative exposure, noise characteristics, and task-related equipment use. Moreover, most studies have examined either audiometric outcomes or occupational noise exposure in isolation, without integrating lifestyle-related risk factors—such as smoking or alcohol consumption—that may interact with noise exposure and further elevate the risk of hearing loss.

Taken together, these findings highlight that NIHL risk in dental professionals, especially dental students is influenced by cumulative exposure, noise characteristics, and equipment use, raising hearing thresholds even if noise levels do not exceed 85 dBA. This study aimed to determine the prevalence of hearing loss, noise characteristics, and risk factors for NIHL among dental professionals.

## 2 Materials and methods

### 2.1 Study design and participants

This cross-sectional analytical study was conducted at a university dental school in southern Thailand. A total of 184 participants, including dentists, dental students, dental assistants, nurses, engineers, technicians, and nonclinical support personnel, were recruited from dental schools.

### 2.2 Data collection and instruments

Data were collected using three components: (1) a self-administered questionnaire, (2) ear examination and audiometric assessment, and (3) workplace noise measurements.

### 2.3 Questionnaire

Participants completed a structured questionnaire designed to obtain demographic, occupational information, use of hearing protection, history of noise exposure, and lifestyle factors, such as tobacco, alcohol, and caffeine consumption.

### 2.4 Ear examination and audiometry

All participants underwent an otoscopic examination. Individuals presenting with current ear disease or prior exposure to sudden noise events were excluded from the study. Subsequently, participants who had not been exposed to noise within 12-h underwent hearing assessment. Pure-tone audiometry was performed, ranging from 250 to 8000 Hz, using headphones in a soundproof room by a certified audiologist. A hearing threshold exceeding 25 dB at any of the tested frequencies, with an audiometric notch (hearing threshold of 3,000, 4,000 or 6,000 Hz being lower than those at 2,000 and 8,000 Hz) was considered abnormal [13].

### 2.5 Noise measurement

Noise measurement data involved both area and personal sampling. An exploration of working areas in the dental hospital was conducted. Sound level meters integrated with a 1/3-octave band analyzer (6.3–20,000 Hz) were set 1.5 m above the floor and placed near the loudest noise source. For personal noise exposure assessments, a noise dosimeter was attached to the collar of each participant throughout their work shift. The device was set at a threshold level of 80 dBA, 8-h criterion level of 85 dBA, and exchange rate of 3 dBA. The parameters recorded included the 8-h time-weighted average (TWA; dBA), maximum noise level (Lmax; dBA), peak impulse noise level (Lpeak; dB), and number of noise peaks detected during the sampling period.

### 2.6 Variables and data analysis

Data analyses were performed using R software (version 4.4.2). Descriptive statistics included percentages and bar graphs, where each bar represents the sound pressure level (SPL) for different frequencies and shows the octave band analysis results obtained from the sound level meters in each working area. The noise level parameters were continuous variables including the 8-h TWA (dBA), Lpeak (dB), Lmax (dBA), and octave-band SPLs (dB), whereas the number of Lpeak events was a discrete variable. For NIHL (normal and abnormal), the distribution of data was determined using the Shapiro-Wilk test and was found to be non-normally distributed (*p* < 0.05).

The general characteristic and lifestyle of participants were on a nominal scale included age, sex, job, caffeine consumption, smoking behavior, and alcohol consumption. Therefore, the chi-squared and Fisher's exact test were used to compare the prevalence of hearing loss between those groups, *p* values <0.05 were considered significant.

The risk factors influencing NIHL in dental personnel included demographic data, lifestyles and noise level in the dental school. Therefore, the independent variables included age, sex, job, caffeine consumption, smoking behavior, alcohol consumption, and noise level such as 8-h-TWA, Lpeak, Lmax, number of even of Lpeak; the dependent variable was NIHL (normal and abnormal). Factors associated with NIHL were evaluated using multiple linear regression, *p* values <0.05 were considered significant. The backward stepwise method was used for the selection of the final model.

### 2.7 Ethical considerations

This study was conducted in accordance with the Declaration of Helsinki and approved by the appropriate Institutional Review Boards of Faculty of Medicine, Prince of Songkla University (REC 67-02309-2), and the Faculty of Dentistry, Prince of Songkla University (EC6703-018). Written informed consent was obtained from all participants.

## 3 Results

### 3.1 Prevalence of hearing loss

A total of 184 participants were enrolled in this study. Overall, these results underscore the multifactorial nature of NIHL risk among dental professionals. Age, work environment, and tobacco use were significant contributors to abnormal hearing thresholds (Table 1). Participants older than 30 years of age showed a significantly higher prevalence of abnormal hearing than younger participants (*p* = 0.016). Those working in maintenance areas and support units demonstrated a significantly higher prevalence of hearing loss than those working in clinical or back-office settings (*p* = 0.019). Tobacco use was significantly associated with abnormal audiometric findings (*p* = 0.004), with 7 out of 9 participants who used tobacco demonstrating hearing impairment. In contrast, gender, occupational role, body mass index (BMI), earphone use, alcohol consumption, caffeine intake, and the use of potentially ototoxic medications were not significantly associated with hearing test results.

**Table 1:** Demographic and characteristics of dental personnel stratified by hearing status (*n* = 184)

Variables	Total (*n* = 184)	Hearing		*p*
		Normal (*n* = 128)	Abnormal (*n*=56)	
Gender	—	—	—	0.137
- Male	40 (21.7)	24 (60)	16 (40)	—
- Female	144 (78.3)	104 (72.2)	40 (27.8)	—
Age (years)	—	—	—	0.016∗
- 20-30	87 (47.3)	68 (78.2)	19 (21.8)	—
- More than 30	97 (52.7)	60 (61.9)	37 (38.1)	—
BMI	—	—	—	0.337
- Less than 25 (non-obesity)	132 (71.7)	95 (72)	37 (28)	—
- ≥ 25 (obesity)	51 (27.7)	33 (64.7)	18 (35.3)	—
Job	—	—	—	0.457
- Dental student	56 (30.4)	44 (78.6)	12 (21.4)	—
- Dentist	41 (22.3)	28 (68.3)	13 (31.7)	—
- Dental assistant and nurse	41 (22.3)	27 (65.9)	14 (34.1)	—
- Nonclinical support personnel	39 (21.2)	24 (61.5)	15 (38.5)	—
- Engineer and technician	7 (3.8)	5 (71.4)	2 (28.6)	—
Workplace	—	—	—	0.019∗
- Back office	9 (4.9)	7 (77.8)	2 (22.2)	—
- Dental workspace	149 (81.0)	109 (73.2)	40 (26.8)	—
- Maintenance area and support unit	26 (14.1)	12 (46.2)	14 (53.8)	—
Working experience (y)	13.00 ± 12.37	11.29 ± 11.19	16.81 ± 14.04	<0.001
Working hour per day	6.87 ± 2.16	7.11 ± 2.12	6.34 ± 2.17	<0.001
Earphone usage	—	—	—	0.625
- No	61 (33.2)	41 (67.2)	20 (32.8)	—
- Yes	123 (66.8)	87 (70.7)	36 (29.3)	—
Tobacco use	—	—	—	0.004∗
- No	175 (95.1)	126 (72)	49 (28)	—
- Yes	9 (4.9)	2 (22.2)	7 (77.8)	—
Alcohol consumption	—	—	—	0.161
- No	91 (49.5)	68 (74.7)	23 (25.3)	—
- Yes	92 (50.0)	60 (65.2)	32 (34.8)	—
Caffeine consumption	—	—	—	0.404
- No	39 (21.2)	25 (64.1)	14 (35.9)	—
- Yes	145 (78.8)	103 (71)	42 (29)	—

∗P < 0.05. BMI, body mass index.

Regarding the prevalence of hearing loss across different job categories, most participants had normal hearing, with dental students showing the highest proportion (78.6%) (Table 2). Unilateral hearing loss was the most frequently observed among dental assistants and nurses (31.7%), and dentists (29.3%). In contrast, bilateral hearing loss was relatively uncommon overall, except among nonclinical support personnel (12.8%) and engineers/technicians (14.3%). While clinical roles in dentistry are associated with some risk of unilateral hearing loss, nonclinical and technical staff may be at greater risk of bilateral hearing impairment.

**Table 2:** Distribution of hearing results by job characteristics (*n* = 184)

Job characteristics	Normal hearing (*n* = 128)	Unilateral hearing loss (*n* = 47)	Bilateral hearing loss (*n* = 9)
Dental student	44 (78.6)	11 (19.6)	1 (1.8)
Dentist	28 (68.3)	12 (29.3)	1 (2.4)
Dental assistant and nurse	27 (65.9)	13 (31.7)	1 (2.4)
Nonclinical support personnel	24 (61.5)	10 (25.6)	5 (12.8)
Engineer and technician	5 (71.4)	1 (14.3)	1 (14.3)

### 3.2 Noise levels and characteristics

The 8-h TWA noise level ranged from 64.33 ± 6.53 to 68.93 ± 5.08 dBA, which is below the 85-dBA occupational exposure limit (Table 3). Dental students and dentists had similar 8-h TWA levels, whereas dental assistants and nurses, nonclinical support personnel, and engineers and technicians were exposed to higher levels. Lmax values were highest among engineers and technicians (103.09 ± 10.06 dBA), indicating peak exposures exceeding other groups. The Lpeak values exhibited a similar pattern, with engineers and technicians exhibiting the highest peaks (131.83 ± 11.76 dB). The number of Lpeak events was notably higher among nonclinical support personnel, dental assistants, nurses, engineers, and technicians, indicating frequent exposure to high-intensity noise. Overall, these findings indicate that while clinical staff experience noise exposure, technical and support roles are subject to higher peak levels and more frequent noise events, potentially increasing their risk of NIHL.

**Table 3:** Noise exposure levels by job characteristics across four parameters (*n* = 184)

Job characteristics	*n* (%)	8-h TWA (dBA)	Lmax (dBA)	Lpeak (dB)	Number of Lpeak
Dental student	56 (30.4)	64.99 ± 4.92	92.90 ± 5.72	128.98 ± 6.77	66.15 ± 31.32
Dentist	41 (22.3)	64.33 ± 6.53	94.65 ± 7.54	127.84 ± 8.76	46.30 ± 32.12
Dental assistant and nurse	41 (22.3)	68.19 ± 5.14	96.10 ± 5.22	124.26 ± 5.89	73.98 ± 35.06
Nonclinical support personnel	39 (21.2)	67.74 ± 6.41	97.00 ± 6.32	126.36 ± 6.43	79.49 ± 56.58
Engineer and technician	7 (3.8)	68.93 ± 5.08	103.09 ± 10.06	131.83 ± 11.76	70.14 ± 26.89

TWA, time-weighted average.

Variations in environmental conditions also contribute to differences in noise levels across workplaces. Dental workspaces, seen in the majority of participants (81.0%), showed moderate noise levels, with an 8-h TWA of 65.84 ± 5.68 dBA and Lpeak of 127.06 ± 7.22 dB (Table 4). Back-office staff experienced similar TWA levels but a slightly lower Lpeak level and number of events. In contrast, staff in maintenance areas and support units were exposed to higher noise levels, with the highest 8-h TWA, Lmax, Lpeak, and number of Lpeak events, indicating more frequent and intense noise exposure. Staff in a technical area or who worked at an ultrasonic cleaning room face a greater risk of NIHL than those in clinical and administrative areas.

**Table 4:** Noise exposure levels by workplaces across four parameters (*n* = 184)

Workplaces	*n* (%)	8-h TWA (dBA)	Lmax (dBA)	Lpeak (dB)	Number of Lpeak
Dental workspaces	149 (81.0)	65.84 ± 5.68	94.41 ± 6.17	127.06 ± 7.22	64.42 ± 37.69
Back office	9 (4.9)	65.38 ± 6.88	95.92 ± 7.52	124.47 ± 7.92	53.89 ± 50.61
Maintenance area and support units	26 (14.1)	69.26 ± 6.01	99.95 ± 7.51	129.01 ± 8.28	83.04 ± 47.59

TWA, time-weighted average.

**Fig. 1:**
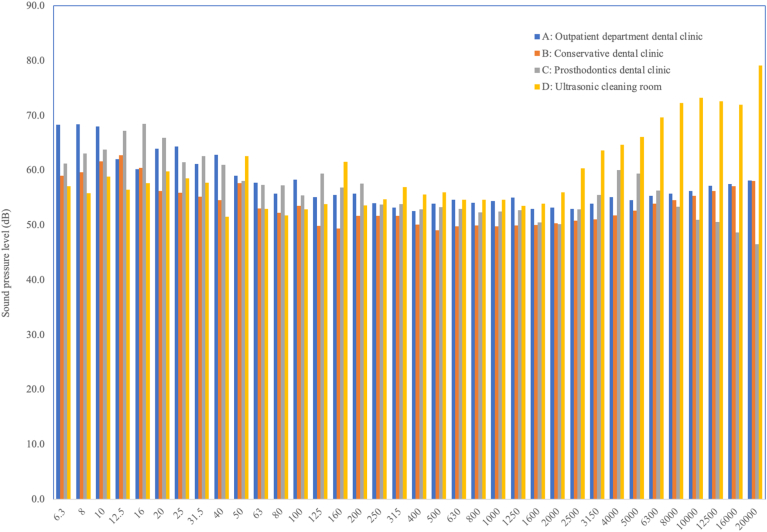
Noise levels and frequency across four workplaces (A: outpatient department dental clinic, B: conservative dental clinic, C: prosthodontics dental clinic, D: ultrasonic cleaning room).

Noise exposure levels vary across workplaces, reflecting the differences in environmental conditions, equipment use, and sound vibrations. Fig. 1 illustrates the overall noise levels and 1/3- octave frequency trends for each setting. In an outpatient dental clinics, the average SPL was 66.41 dBA, with peak noise frequencies below 25 Hz reaching up to 70 dB. The conservative dental clinic had a slightly lower 8-h equivalent continuous average level (Leq) of 63.9 dBA, with peaks concentrated in the 5000–20,000-Hz range at approximately 60 dB. In the prosthodontics dental clinic, the Leq was 66.41 dBA, with noise peaks around 4000, 5000, and 6300 Hz and levels reaching 65 dB. In contrast, the ultrasonic cleaning room showed the highest Leq of 78.19 dBA, with peak frequencies above 6300 Hz and levels reaching 80 dB. These results highlight that the overall intensity and frequency characteristics of noise differ by workplace, with the ultrasonic cleaning room presenting both higher levels and higher-frequency peaks, potentially increasing the risk of NIHL.

### 3.3 Risk factors for hearing loss

The analysis of risk factors (Table 5) demonstrated that age, lifestyle behaviors, and noise exposure characteristics significantly influenced the likelihood of NIHL. The logistic regression model demonstrated that, compared to dental students, other job categories did not show significant associations with NIHL after adjustment (*p* = 0.664). However, participants aged over 30 years had a nearly three-fold higher prevalence of NIHL than those aged 20–30 years (adjusted odds ratio [adjOR] = 2.93, 95% confidence interval [CI]: 1.01–8.49, *p* = 0.037). Lifestyle factors were also significant: participants who consumed tobacco had an increased prevalence (adjOR = 5.81, 95% CI: 1.01–33.28, *p* = 0.034) and those who consumed alcohol showed more than a three-fold higher risk (adjOR = 3.21, 95% CI: 1.33–7.74, *p* = 0.007). In contrast, caffeine consumption was associated with a reduced risk of NIHL (adjOR = 0.33, 95% CI: 0.13–0.85, *p* = 0.021). Lpeak levels showed a nonsignificant association (adjOR = 1.05, 95% CI: 1.0–1.1, *p* = 0.061). Overall, these findings highlight that, beyond occupational roles, individual characteristics and lifestyle behaviors play critical roles in the risk of hearing loss.

**Table 5:** Risk factors associated with noise induced hearing loss (NIHL) (*n* = 184)

Variables	Crude OR (95% CI)	Adjusted OR (95% CI)	*p*
Jobs (ref. = dental student)			
- Dentist	1.78 (0.69,4.59)	0.91 (0.23,3.57)	0.664
- Dental assistant and nurse	2.07 (0.82, 5.22)	1.88 (0.52,6.78)	—
- Nonclinical support personnel	2.33 (0.92, 5.93)	1.08 (0.27, 4.34)	—
- Engineer and technician	1.6 (0.27,9.37)	0.79 (0.1,6.47)	—
Age (ref. = 20-30 years)	—	—	0.037∗
- More than 30 years	2.24 (1.15,4.37)	2.93 (1.01,8.49)	—
Tobacco use (ref. = No)	—	—	0.034∗
- Yes	9.51 (1.91, 47.46)	5.81 (1.01, 33.28)	—
Alcohol consumption (ref. = No)	—	—	0.007∗
- Yes	1.57 (0.82-3.01)	3.21 (1.33,7.74)	—
Caffeine consumption (ref. = No)	—	—	0.02∗
- Yes	0.68 (0.32,1.45)	0.33 (0.13,0.85)	—
Lpeak (cont.)	1.03 (0.99, 1.07)	1.05 (1.0, 1.1)	0.061

∗P < 0.05. CI, confidence interval; OR, odds ratio.

## 4 Discussion

In this study, participants were recruited from both clinical-year dental students and dental hospital personnel including dentists, senior dental students (5th–6th year), dental assistants, dental technicians, and other support staff such as central supply workers, engineers, and maintenance personnel. Recruitment followed a stratified sampling approach based on job categories within each department to ensure representation across occupational roles. Because clinical-year dental students constitute the largest population within the faculty and demonstrated strong interest and availability to participate, they formed the largest subgroup in our sample (30.4%). This study highlights the importance of effective hearing protection for dental students in their career development as they are likely to experience prolonged occupational noise exposure.

Although most participants demonstrated normal hearing thresholds, unilateral hearing loss was frequently found among clinical personnel. This pattern may be related to proximity to dental instruments and asymmetric exposure to noise during clinical procedures. In dental practices, primary sound sources such as high-speed handpieces and suction devices, are often located closer to one ear than the other, resulting in greater unilateral exposure. When noise sources are in close proximity to one side, significant asymmetry in hearing thresholds may occur [14]. Further, left-side hearing thresholds are significantly lower in dental assistants due to the presence of noise from instruments positioned on the left side [15]. In contrast, nonclinical and technical staff members, who are typically exposed to broader environmental or machine-generated noise, demonstrated a higher prevalence of bilateral hearing loss. This finding is consistent with the noise assessment results, which indicate that more intense peak levels and diffuse exposure patterns likely affect both ears simultaneously.

This study examined noise levels in a dental school and found that 8-h TWAs ranged from 64.33 ± 6.53 to 68.93 ± 5.08 dBA, which is below the United States National Institute of Occupational Safety and Health (NIOSH) occupational exposure limit of 85 dBA [16]. These findings are consistent with a previous report on noise in dental clinics and laboratories, which showed median noise levels of 62.2 and 66.2 dBA, respectively [8]. Similarly, noise levels during practical sessions of fixed prosthodontics were reported to be between 69.35 and 72.06 dBA [17]. Notably, the workplace environment and equipment use strongly influence the intensity and frequency of noise exposure, potentially affecting the risk of NIHL among staff. Although the average exposure levels in dental settings remained below the NIOSH 8-h limit, elevated Lpeak values and repeated high-intensity noise events were observed, particularly in the ultrasonic cleaning room. These peak exposures likely play a key role in the development of hearing loss even when the average noise levels are within the recommended limits.

Regarding the impact of peak noise, the highest Lpeak of 131.83 dBA was observed in the engineer and technician groups, approaching the NIOSH ceiling limit of 140 dBA for instantaneous exposure [16]. This finding aligns with the observed higher prevalence of hearing loss in nonclinical workers, suggesting that intense short-duration noise events contribute substantially to auditory risk. Mohammad et al. reported a 23%–28% increase in hearing loss among individuals exposed to impact noise compared to that in individuals exposed to continuous noise [18]. Furthermore, in complex noise environments with high-impact noise exposure, NIHL may begin to occur when the 8-h Leq exceeds 70 dBA [19], highlighting that, even when the average exposure remains below the recommended limits, impact noise and frequent peak events can still pose a significant threat to hearing. This study provided detailed 1/3-octave noise frequency band information for individual workplaces, which can be used to inform engineering controls.

These findings reinforce the idea that NIHL in dental environments is influenced not only by occupational noise exposure but also by individual and lifestyle factors. The main risk factors associated with hearing loss were age and tobacco and alcohol consumption, suggesting that both cumulative physiological changes and modifiable behaviors contribute to susceptibility. Age emerged as an important factor, with participants older than 30 years showing significantly higher odds of abnormal hearing, which is consistent with age-related vulnerability to noise damage. These results are consistent with previous reports demonstrating that age-related auditory decline interacts with occupational exposure and accelerates threshold shifts among older workers [20]. Furthermore, systematic reviews of hearing loss in dental professionals showed a positive association between age and NIHL in 6 out of 8 studies, especially in those aged >40 years [21]. Age and occupational noise converged through shared mechanisms, including cumulative cochlear hair cell loss and reduced auditory system repair, thereby accelerating SNHL. In addition to age-related susceptibility, lifestyle behaviors, such as tobacco and alcohol consumption, have also been implicated as important contributors to NIHL, emphasizing the multifactorial risk of NIHL in dental professionals.

Tobacco use has been shown to independently increase the risk of SNHL, with proposed mechanisms, including reduced cochlear blood flow, hypoxia, and oxidative stress that exacerbating the damaging effects of noise exposure. In this study, nearly 78% of participants who used tobacco demonstrated abnormal hearing thresholds, a proportion markedly higher than that of those who did not use tobacco, emphasizing that tobacco use is an important cofactor. Similarly, tobacco use has been identified as an independent risk factor for SNHL [17]. A meta-analysis of 27 studies reported that people who use tobacco had more than twice the risk of NIHL than those who do not [22]. The mechanisms linking tobacco use and NIHL remain unclear; however, nicotine and other tobacco components may exert ototoxic effects by elevating carboxyhemoglobin levels or decreasing the cochlear blood supply, thereby decreasing the resistance of hair cells to noise-induced damage [23,24]. Moreover, nicotine-like receptors have been identified in cochlear hair cells, indicating that nicotine may directly impair hair cell function through ototoxic mechanisms [25]. These findings provide consistent evidence that tobacco use is an important risk factor that increases the incidence of NIHL and highlight the need to address lifestyle risks alongside occupational exposure.

A similar concern arises with alcohol consumption, which has also been associated with hearing impairment. In this study, alcohol consumers exhibited more than a three-fold higher risk of abnormal hearing. This finding is consistent with previous evidence linking alcohol use to NIHL. Alcohol intake appears to increase the risk of hearing loss, indicating that its influence may be more strongly related to NIHL [26,27]. Moreover, the risk of hearing loss appears to increase with the level of alcohol consumption. The hazard ratio for hearing loss was significantly higher in the heavy consumption group than in the moderate consumption group [27]. However, the biological mechanisms underlying the combined effects of alcohol consumption and noise exposure on hearing remain unclear. This effect may be associated with damage to the vascular and auditory nerves caused by alcohol and noise exposure [28,29]. The importance of considering both alcohol consumption and noise exposure should be highlighted when assessing the risk factors for hearing loss and developing preventive strategies.

In contrast to alcohol consumption, caffeine consumption appeared to protect against NIHL, with an adjusted odds ratio of 0.33. These findings suggest that caffeine intake may reduce the risk of NIHL, potentially by improving cochlear blood flow and antioxidant activity. However, the majority of experimental studies have reported contradictory results. A large cross-sectional analysis of adults in the USA found that high caffeine intake was significantly associated with an increased risk of speech-frequency hearing loss, particularly in individuals under 65 years of age [30]. These findings highlight the inconsistency in the current evidence and suggest that the effects of caffeine on hearing may vary depending on exposure conditions and age. Furthermore, experimental data from a guinea pig model demonstrated that animals exposed to both caffeine and noise exhibited persistent threshold shifts, whereas those exposed to noise alone exhibited near-complete recovery, indicating that caffeine impaired auditory recovery after temporary threshold shifts [31]. Thus, while caffeine has been linked to a reduced risk of NIHL in certain populations, biological evidence indicates that it may interfere with auditory recovery following temporary threshold shifts, highlighting the need for further investigations of its complex effects on hearing health. Therefore, tobacco smoke, alcohol, and caffeine consumption should be investigated more thoroughly in terms of the total amount of consumed and duration of use to establish a dose-response relationship.

Age, lifestyle behaviors, and noise exposure significantly influence hearing outcomes. The findings of this study indicate that lifestyle factors can differentially influence auditory health, emphasizing the need for preventive programs that combine workplace noise control with health promotion strategies. Specifically, those older than 30 years and those who use alcohol or tobacco were at higher risk, whereas caffeine consumption showed a protective association. However, this study has some limitations, including the absence of baseline audiometry, lack of a control group to isolate age-related high-frequency loss, and lack of quantitative assessment of dose-dependent risk factors, such as alcohol and caffeine. The related factors and hearing loss was reported in this study. This cross-sectional, analytical study has limitation in establishing causation of relationship; therefore, the noise exposure should be experience at least one year. The dental students were prone to noise exposure from the 4th year of their clinical training. In addition, the longitudinal study to follow-up on the hearing loss in young dentists should be investigated. However, the study's key strength lies in its comprehensive assessment of both environmental and personal noise exposure, coupled with objective audiometric data. This study is among the first to investigate hearing loss in dental personnel using this approach and has revealed a high prevalence of NIHL across job categories. These results support the need for early detection programs and targeted preventive interventions in this professional group. This study also highlights the importance of effective hearing protection for dental students in their career development as they are likely to experience prolonged occupational noise exposure.

This study demonstrated that occupational noise exposure and lifestyle factors play important roles in the development of NIHL among dental personnel. Older age, tobacco use, and alcohol consumption were associated with a higher risk of NIHL, whereas caffeine consumption showed a protective association in certain populations, although experimental evidence suggests that caffeine may impair auditory recovery. The borderline association between peak noise levels and NIHL further emphasizes the hazard of impact noise. By integrating audiometric findings with objective noise measurements and lifestyle-related risk factors, this study offers a more comprehensive characterization of NIHL risk in dental personnel than previously available. These findings highlight the need for preventive strategies that integrate engineering controls to reduce noise exposure with health promotion initiatives that address modifiable lifestyle risks. Early detection using extended high-frequency audiometry and targeted interventions are essential to protect hearing and reduce the burden of NIHL in this vulnerable group.

## CRediT authorship contribution statement

**Thitiworn Choosong:** Writing – review & editing, Writing – original draft, Methodology, Funding acquisition, Data curation, Conceptualization. **Sittichok Teawanan:** Methodology, Data curation. **Ramida Dindamrongkul:** Writing – review & editing, Writing – original draft, Resources, Methodology, Data curation. **Wandee Khaimook:** Data curation. **Sasithorn Srimeechai:** Conceptualization. **Paranicha Bunruang:** Writing – original draft. **Phattaranun Phiboon:** Writing – original draft. **Wannadee Wacharachaipong:** Writing – original draft.

## Informed consent statement

Informed consent was obtained from all participants involved in the study.

## Ethics statement

The study was conducted in accordance with the Declaration of Helsinki and approved by the Institutional Review Boards of the Faculty of Medicine, Prince of Songkla University (REC 67-02309-2), and the Faculty of Dentistry, Prince of Songkla University (EC6703-018).

## Availability of data and materials

Data will be made available upon request from authors.

## Statement on the use of AI tools

During the preparation of this work, the authors used ChatGPT (OpenAI) solely to improve grammar and language readability. After using this tool, the authors carefully reviewed and edited the content as needed and take full responsibility for the content of the publication.

## Funding

This research was supported by the National Science, Research, and Innovation Fund (NSRF) and 10.13039/501100004508Prince of Songkla University, Thailand [grant no. 4693399].

## Conflict of interest

All authors have no conflicts of interest to declare.
